# Supplementary material for: Functional liver imaging score (FLIS): A prognostic biomarker for acute-on-chronic liver failure and liver-related mortality
Source: JHEP Rep. 2026 Jun 17;8(8):101928. doi: 10.1016/j.jhepr.2026.101928 (PMC13396738; doi:10.1016/j.jhepr.2026.101928)
Supplement: Multimeda component 1 [file mmc1.pdf]

Functional liver imaging score (FLIS): A prognostic biomarker for acute-on-chronic liver failure and liver-related mortality

Sarah Poetter-Lang, Lorenz Balcar, Ahmed Ba-Ssalamah, Nina Bastati, Raphael Ambros, Antonia Kristic, Julia Krawanja, Katharina Pomej, Benedikt Simbrunner, Georg Semmler, Viktor Schmidbauer, Svitlana Pochepnia, Daniel Sobotka, Jacqueline C. Hodge, Ulrike Attenberger, Michael Trauner, Thomas Reiberger, Lucian Beer, Mattias Mandorfer

Table of contents

Table S1……………………………………………………………………………2

Table S2……………………………………………………………………………3

Table S3……………………………………………………………………………4

Table S4……………………………………………………………………………5

Table S1……………………………………………………………………………6

Fig. S1……………………………………………………………………………..…..7

Table S1. Comparison of imaging characteristics of patients with vs without ACLF at study inclusion.

Imaging characteristics, mean (SD)	No ACLF at baseline, n=210	ACLF at baseline, n=6
FLIS	4.3 (1.9)	1.1 (3.2)
RLE	84.6 (47.8)	56.1 (25.2)
REB	7.2 (30.2)	1.1 (0.7)
LPC	1.6 (0.6)	1.2 (0.3)
ACLF, acute-on-chronic liver failure; FLIS, functional liver imaging score; LPC, liver to portal vein ratio; REB, relative enhancement ratio of the biliary system; RLE, relative liver enhancement; SD, standard deviation		

Table S2. MRI parameters for 3T images.

Sequence	Section Thickness (mm)	TR (msec)	TE (msec)	FOV (mm)	Phase Direction	Flip Angle
GRE-T1 (flash 2D) in-phase	5	130	2.46	350	AP	70
GRE-T1 (flash 2D) opposed-phase	5	131	3.69	350	AP	70
T1 VIBE SPAIR axial	1.7	2.67	0.97	430	AP	13
T1 VIBE SPAIR coronal	2	2.6	0.92	500	RL	13
T2 Haste coronal	4.5	805	76	450	RL	141
DWI axial TSE-EP	6	1700	73	380	AP	---
T2 MRCP 2D-HASTE	45	5500	454	380	RL	180
T2 MRCP 3D-SPACE	0.9	2400	707	350	RL	variable
T2 Haste axial fs.	5	1800	150	400	AP	150

2D, two-dimensional; FOV, field of view; fs, fat saturation; GRE, gradient echo; HASTE, half-fourier-acquired single-shot turbo spin echo; MRCP, magnetic resonance cholangiopancreatography; MRI, magnetic resonance imaging; SPAIR, spectral attenuated inversion recovery; TE, echo time; TR, repetition time; TSE, turbo spin echo; VIBE, volumetric interpolated breath-hold examination

Table S3. Inter-observer variability of semi-quantitative and quantitative gadoxetic acid scores.

Variable	ICC	95%CI
RLE	0.95	0.88-0.98
LPC	0.91	0.88-0.93
REB	0.82	0.75-0.87
FLIS	0.90	0.82-0.94

CI, confidence interval; FLIS, functional liver imaging score; ICC, intraclass correlation coefficient; LPC, liver to portal vein ratio; REB relative enhancement ratio of the biliary system; RLE, relative liver enhancement

Table S4. Summary of clinical outcomes during follow-up.

	AD, n=49	Clinically stable ACLD, n=161	Decompensated cirrhosis, n=100	Compensated ACLD, n=110
Acute-on-chronic liver failure, n (%)	20 (41%)	29 (18%)	33 (33%)	16 (15%)
Liver transplantation, n (%)	5 (10%)	11 (7%)	8 (8%)	8 (7%)
Deaths, n (%)	25 (51%)	23 (14%)	37 (37%)	11 (10%)
Liver-related deaths, n (%)	23 (47%)	22 (14%)	34 (34%)	11 (10%)
Acute-on-chronic liver failure or liver-related death, n (%)	37 (76%)	47 (29%)	60 (60%)	24 (22%)
Median time to acute-on-chronic liver failure/liver-related death; months (95%CI)	8.0 (3.1-31.3)	34.6 (30.1-41.3)	32.7 (12.3-40.0)	38.8 (30.1-45.6)

ACLD, advanced chronic liver disease; AD, acute decompensation; CI, confidence interval

Table S5. Comparison of organ dysfunctions/failures according to the CLIF-SOFA score in patients developing ACLF during follow-up between FLIS 0-3 vs. 4-6.

Patient characteristics, n (%)	FLIS 4-6, n=29	FLIS 0-3, n=28	p-value
**Liver**			
Bilirubin <6 mg/dL	16 (72.7%)	5 (18.5%)	
Bilirubin 6-12 mg/dL	1 (4.5%)	6 (22.2%)	**<0.001**
Bilirubin ≥12 mg/dL	5 (22.7%)	16 (59.3%)	
**Kidney**			
Creatinine <2 mg/dL	7 (31.8%)	7 (25.9%)	
Creatinine 2.0-3.5 mg/dL	10 (45.5%)	15 (55.6%)	0.781
Creatinine ≥3.5 mg/dL or RRT	5 (22.7%)	5 (18.5%)	
**Brain**			
West-Haven 0	11 (50.0%)	11 (40.7%)	
West-Haven I-II	5 (22.7%)	11 (40.7%)	0.397
West-Haven III-IV	6 (27.3%)	5 (18.5%)	
**Coagulation**			
INR <2.0	16 (72.7%)	16 (59.3%)	
INR 2.0-2.5	5 (17.2%)	9 (32.1%)	0.418
INR ≥2.5	3 (10.3%)	2 (7.1%)	
**Circulation**			
MAP ≥70 mmHg	14 (63.6%)	17 (63.0%)	
MAP <70 mmHg	3 (13.6%)	3 (11.1%)	0.944
Vasopressors	5 (22.7%)	7 (25.9%)	
**Respiration**			
PaO_2/FiO_2 >300	18 (81.8%)	24 (88.9%)	
PaO_2/FiO_2 200-300	1 (4.5%)	2 (7.4%)	0.428
PaO_2/FiO_2 <200	3 (13.6%)	1 (3.7%)	
**Minimal ACLF grade**			
1a	6 (27.3%)	3 (11.1%)	
1b	4 (18.2%)	5 (18.5%)	0.519
2	9 (40.9%)	15 (55.6%)	
3	3 (13.6%)	4 (14.8%)	
**Maximum ACLF grade**			
1a	5 (22.7%)	1 (3.7%)	
1b	1 (4.5%)	2 (7.4%)	0.240
2	10 (45.5%)	16 (59.3%)	
3	6 (27.3%)	8 (29.6%)	

ACLF, acute-on-chronic liver failure; FLIS, functional liver imaging score

Fig. S1

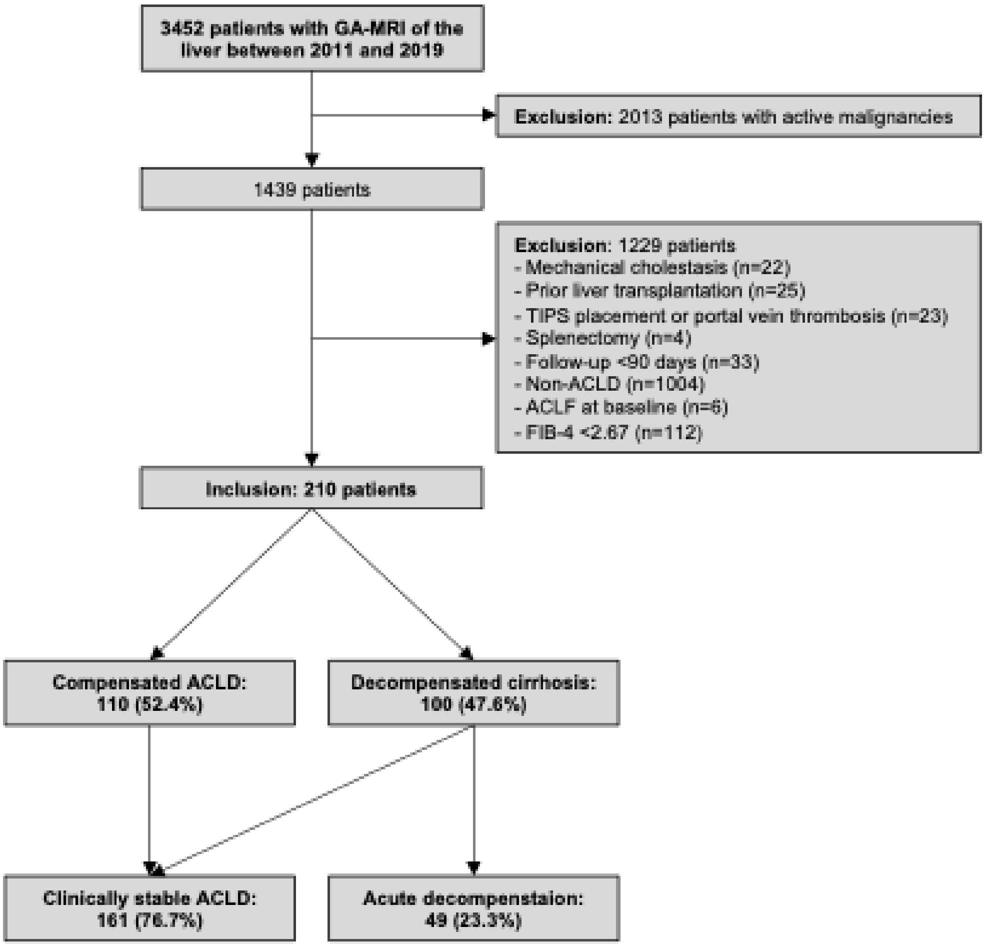

Fig. S1. Study flowchart.

Abbreviations: ACLF, acute-on-chronic liver failure; ACLD, advanced chronic liver disease; AD, acute decompensation; cACLD, compensated advanced chronic liver disease; GA, gadoxetic acid; MRI, magnetic resonance imaging; TIPS, transjugular intrahepatic portosystemic shunt
