# Supplementary material for: Functional liver imaging score (FLIS): A prognostic biomarker for acute-on-chronic liver failure and liver-related mortality
Source: JHEP Rep. 2026 Jun 17;8(8):101928. doi: 10.1016/j.jhepr.2026.101928 (PMC13396738; doi:10.1016/j.jhepr.2026.101928)
Supplement: Multimedia component 4 [file mmc4.pdf]

JHEP|Reports

Functional liver imaging score (FLIS): A prognostic biomarker for acute-on-chronic liver failure and liver-related mortality

Authors

Sarah Poetter-Lang, Lorenz Balcar, Ahmed Ba-Ssalamah, …, Thomas Reiberger, Lucian Beer, Mattias Mandorfer

Correspondence

ahmed.ba-ssalamah@meduniwien.ac.at (A. Ba-Ssalamah).

Graphical abstract

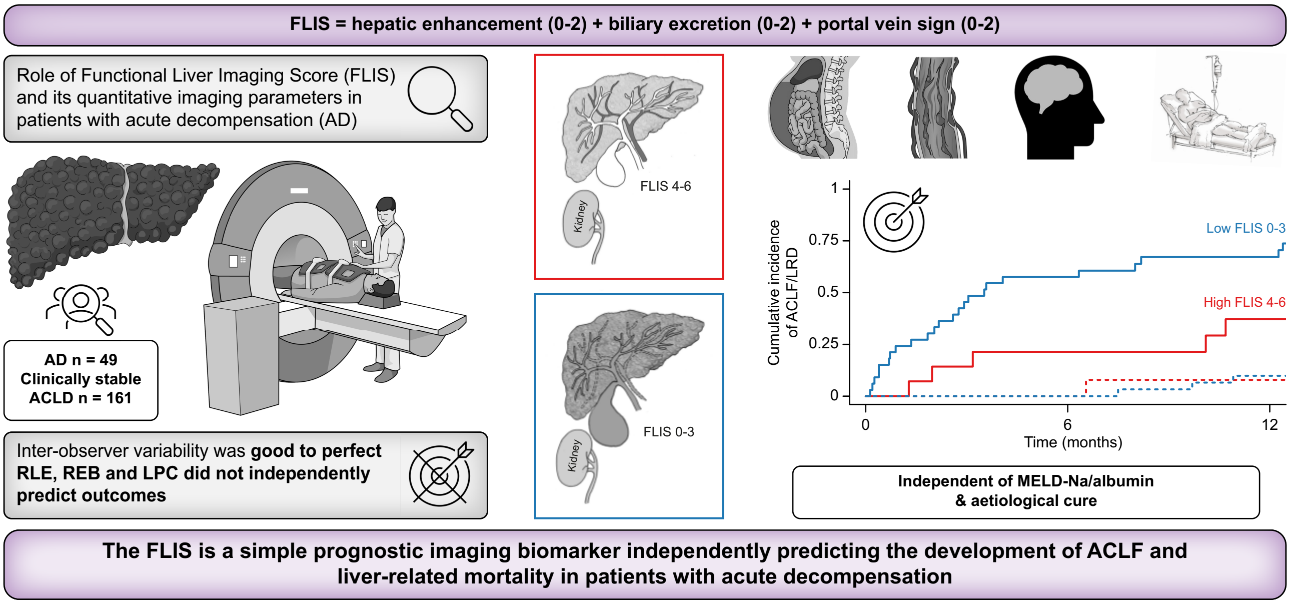

Highlights:

- FLIS independently predicts acute-on-chronic liver failure and liver-related death in acute decompensation.
- FLIS is semi-quantitative and easy to calculate.
- Unlike its quantitative MRI components, FLIS provides incremental prognostic information.

Impact and implications:

The visual, semi-quantitative functional liver imaging score (FLIS), derived from gadoxetic acid-enhanced MRI, assesses liver function. We found that FLIS predicts acute-on-chronic liver failure and liver-related mortality in patients with acutely decompensated cirrhosis, independent of established risk scores. Conversely, quantitative MRI parameters did not add prognostic value. Thus, FLIS-based risk stratification may help identify patients who could benefit from intensified monitoring or timely liver transplant evaluation.

https://doi.org/10.1016/j.jhepr.2026.101928

© 2026 The Authors. Published by Elsevier B.V. on behalf of European Association for the Study of the Liver (EASL). This is an open access article under the CC BY license (http://creativecommons.org/licenses/by/4.0/).

JHEP|Reports

Functional liver imaging score (FLIS): A prognostic biomarker for acute-on-chronic liver failure and liver-related mortality☆

Sarah Poetter-Lang[1,†], **Lorenz Balcar**[2,3,†], Ahmed Ba-Ssalamah[1,*], Nina Bastati[1], Raphael Ambros[4], Antonia Kristic[1], Julia Krawanja[5], Katharina Pomej[2,3], Benedikt Simbrunner[2,3], Georg Semmler[2,3], Victor Schmidbauer[1], Svitlana Pochepnia[1], Daniel Sobotka[6,7], Jacqueline C. Hodge[1], Ulrike Attenberger[1], Michael Trauner[3], Thomas Reiberger[2,3], Lucian Beer[1,‡], Mattias Mandorfer[2,3,‡]

JHEP Reports **2026**. vol. 8 | 1–10

Check for updates

Background & Aims: The functional liver imaging score (FLIS; range 0–6, with higher scores indicating better liver function), derived from gadoxetic acid-enhanced MRI, is a prognostic imaging biomarker in advanced chronic liver disease (ACLD). Our aim was to investigate whether semi-quantitative FLIS and quantitative imaging parameters, namely relative liver enhancement (RLE), relative enhancement ratio of the biliary system (REB), and liver-to-portal vein contrast ratio (LPC), predict acute-on-chronic liver failure (ACLF; a syndrome defined by extrahepatic organ failure and high short-term mortality) and liver-related mortality in acute decompensation (AD), the main at-risk population.

Methods: We included 210 patients with ACLD who underwent GA-MRI-derived semi-quantitative FLIS assessment, in whom RLE, REB, and LPC were also computed by two independent, experienced radiologists. Patients were stratified into clinically stable ACLD (including compensated or non-acutely decompensated disease) and AD. The prognostic value of semi-quantitative FLIS and quantitative GA-MRI parameters for ACLF/liver-related death was evaluated using competing risk regression analyses, with liver transplantation considered a competing event.

Results: FLIS was lower in AD (*vs.* clinically stable ACLD). Furthermore, low FLIS was an independent risk factor for ACLF development/liver-related death in AD (adjusted subdistribution hazard ratio [aSHR]: 2.37 [95% CI 1.08–5.17], p = 0.031; adjusted for MELD-Na, albumin, and etiological cure), but not in clinically stable ACLD (aSHR: 1.58 [95% CI 0.73–3.45], p = 0.250). Conversely, while RLE, REB, and LPC distinguished between AD and clinically stable ACLD (p <0.001), they did not predict ACLF/liver-related death.

Conclusions: The FLIS is a simple prognostic imaging biomarker in acute decompensation that predicts ACLF and liver-related mortality. FLIS-based risk stratification may help identify patients who could benefit from intensified monitoring or timely liver transplant evaluation.

© 2026 The Authors. Published by Elsevier B.V. on behalf of European Association for the Study of the Liver (EASL). This is an open access article under the CC BY license (http://creativecommons.org/licenses/by/4.0/).

Introduction

Acute-on-chronic liver failure (ACLF) is a life-threatening systemic condition in the setting of acute decompensation (AD) of advanced chronic liver disease (ACLD)/cirrhosis, with 28- and 90-day mortality rates of around 30% and 63%, respectively.[1,2] While portal hypertension is a key driver of AD in ACLD, a burst of systemic inflammation is hypothesised to trigger ACLF, which then perpetuates and results in multi-organ failure.[3] ACLF is recognised as a dynamic entity.[4] Current management involves treatment of precipitating factors and extrahepatic organ dysfunction/failure. However, in patients with worsening ACLF, orthotopic liver transplantation represents the only curative treatment option. As donor organs are scarce, early detection of ACLF at reversible stages becomes imperative.[1] However, the ideal strategy to reduce the burden of life-threatening ACLF would be its prevention. A recent study specifically designed for this purpose observed an event rate of only approximately 13% over a 12-month period,[5] indicating that biomarkers to enrich trials with patients at increased risk are needed. While there has been considerable progress in the field of blood-based biomarkers in decompensated cirrhosis, imaging-based biomarkers,[6] which have revolutionised the field of compensated ACLD,[7] remain understudied, despite the fact that imaging is routinely performed due to the recommended biannual hepatocellular carcinoma screening.

Since hepatic dysfunction is a key determinant of outcomes in ACLD, evaluation of the prognostic abilities of established

☆ Given their role as Associate Editor, Mattias Mandorfer had no involvement in the peer-review of this article and had no access to information regarding its peer-review. Full responsibility for the editorial process for this article was delegated to the Guest Editor Annalisa Berzigotti and Editor-in-Chief Josep M Llovet.

* Corresponding author. Address: Medical University Vienna, General Hospital of Vienna (AKH), Department of Biomedical Imaging and Image-guided Therapy. Waehringer Guertel 18-20, A-1090 Vienna, Austria; Tel.: 00431 40400 48930, fax: 00431 40400 48940.

E-mail address: ahmed.ba-ssalamah@meduniwien.ac.at (A. Ba-Ssalamah).

† Shared first authorship

‡ Share senior authorship

https://doi.org/10.1016/j.jhepr.2026.101928

imaging biomarkers seems a promising approach.[8,9] Although MRI is not universally available in ACLD, the functional liver imaging score (FLIS), a semi-quantitative biomarker derived from gadoxetic acid (GA)-enhanced MRI, requires neither dedicated hardware/software nor complex calculations, and has previously been shown to be independently associated with liver-related outcomes,[9] including ACLF. However, the latter study did not differentiate between AD and other clinically distinct populations. Thus, we cannot rule out that the association between FLIS and ACLF development was confounded by the presence/absence of AD.

Therefore, our goal was to evaluate the prognostic value of FLIS and its quantitative imaging parameters, namely relative liver enhancement (RLE), relative enhancement ratio of the biliary system (REB), and liver-to-portal vein contrast ratio (LPC) for ACLF development in 210 patients, stratified by clinical context.

Patients and methods

Study population

Our inclusion criteria were (i) GA-enhanced MRI including 20-minute hepatobiliary phase images obtained between 2011 and 2019 using a standard examination protocol, (ii) ACLD, (iii) follow-up ≥90 days (including imaging and/or clinical visit), and (iv) all subsequent laboratories acquired within 2 weeks of the MRI examination: platelet count, white blood cell count, prothrombin time, international normalised ratio, albumin, bilirubin, alkaline phosphatase, alanine aminotransferase, aspartate aminotransferase, creatinine, and sodium.

The exclusion criteria were: (i) current or prior malignancy; (ii) mechanical cholestasis; (iii) prior orthotopic liver transplantation; or (iv) transjugular intrahepatic portosystemic shunt placement; (v) portal vein thrombosis; (vi) splenectomy; or (vii) ACLF at baseline. GA-MRI-based parameters in patients with (*i.e.* finally excluded from the main analysis) *vs*. without ACLF at baseline are shown in the Supplementary materials (Table S1).

Subgroups of patients included in this study have previously been reported in three studies evaluating the prognostic value of FLIS for predicting hepatic decompensation and mortality in patients with ACLD,[10] as well as to evaluate the changes in GA uptake after hepatitis C virus eradication.[11]

Clinical data

Electronic health records (in- and outpatient documentation within our centre, other Viennese hospitals, as well as national

Table 1. Patient characteristics.

Patient characteristics	Clinically stable ACLD, n = 161	AD, n = 49	*p* value
Age, years	50 ± 15	59 ± 19	0.481
Sex			
Male	104 (65%)	37 (75%)	
Female	57 (35%)	12 (25%)	0.104
Indication			
Query hepatic nodules	127 (79%)	40 (82%)	0.683
Query biliary obstruction	8 (5%)	2 (4%)	0.523
Work-up of PSC/PBC	7 (4%)	2 (4%)	0.452
Work-up of non-cholestatic ACLD	3 (2%)	—	—
Other/unknown	19 (10%)	5 (10%)	0.913
Aetiology			
ALD	35 (22%)	19 (39%)	**0.036**
HCV	54 (34%)	7 (14%)	0.352
HBV	11 (7%)	3 (6%)	0.634
PBC/PSC	16 (10%)	3 (6%)	0.634
MASLD	19 (11%)	2 (4%)	**0.035**
AIH	9 (6%)	5 (10%)	0.645
Genetic	5 (3%)	—	—
Cryptogenic	10 (6%)	10 (21%)	0.436
Other	2 (1%)	—	—
Varices (yes)	136 (78%)	48 (98%)	**<0.001**
CTP stage			
A	105 (65%)	7 (14%)	**<0.001**
B	48 (30%)	24 (49%)	**<0.001**
C	8 (5%)	18 (37%)	**<0.001**
MELD-Na, points	9 (7)	16 (11)	**<0.001**
Platelet count, G/L	92 (62)	107 (97)	0.073
Albumin, g/L	39 (9)	31 (9)	**<0.001**
Bilirubin, mg/dl	1.2 (1.3)	2.72 (4.56)	**<0.001**
INR	1.3 (0.3)	1.4 (0.3)	**0.005**
Creatinine, mg/dl	0.82 (0.32)	0.86 (0.6)	0.051
Sodium, mmol/L	137 (0)	136 (4)	0.230
ALP, U/L	103 (78)	102 (71)	0.911
GGT, U/L	87 (161)	64 (113)	0.503
AST, U/L	51 (41)	46 (53)	0.907
ALT, U/L	37 (36)	28 (33)	**0.02**

Continuous variables were compared using the Mann–Whitney U test or unpaired t test, as appropriate. Categorical variables were compared using Fisher's exact test. Bold type indicates statistically significant values (p <0.05). ACLD, advanced chronic liver disease; AIH, autoimmune hepatitis; ALT, alanine aminotransferase; ALP, alkaline phosphatase; ALD, alcohol-related liver disease; AST, aspartate aminotransferase; CTP, Child-Turcotte-Pugh; GGT, gamma-glutamyltransferase; INR, international normalised ratio; MASLD, metabolic dysfunction-associated steatotic liver disease; MELD, model for end-stage liver disease; PBC, primary biliary cholangitis; PSC, primary sclerosing cholangitis.

health records) were reviewed by M.Ds. (L.Ba, J.K., K.P., B.S., and G.S.) under the supervision of specialists in gastroenterology/hepatology (T.R., and M.M.) with >10 years of experience. The investigators reviewing the clinical data were blinded to imaging data. Demographic and clinical data are shown in Table 1.

ACLD definition and severity classification

ACLD was defined by a hepatic venous pressure gradient (HVPG) ≥6 mmHg and/or indicators of clinically significant portal hypertension (*i.e.* presence of varices on endoscopy, portosystemic collaterals on imaging, or ascites) and/or histologic signs of cirrhosis, and/or an Fibrosis-4 score (FIB-4) ≥2.67,[12] which complements liver stiffness measurement in the diagnosis of cACLD according to Baveno VII[13] and has been found to be roughly equivalent to ≥15 kPa in terms of hepatic decompensation risk in a previous study from our centre.[14] The following formula was used to calculate the FIB-4 score: FIB-4 = age (years) × aspartate aminotransferase (U/L)/[platelet count (10^9/L) × alanine aminotransferase (U/L)].[12]

Patients with no history of or current hepatic decompensation (as defined by Baveno VII[13]) were classified as having compensated ACLD (cACLD), whereas those with a history of or current hepatic decompensation were classified as having decompensated cirrhosis. Patients with a recent (*i.e.* within 28 days) acute hepatic decompensation requiring a liver-related hospitalisation at the time of GA-MRI examination were classified as having AD.[15] Clinically stable patients with ACLD included those with cACLD as well as those with decompensated cirrhosis not meeting AD criteria.

Definition of ACLF

ACLF was defined according to the EASL-CLIF criteria.[2] Respective organ dysfunctions/failures were defined according to the CLIF-SOFA score.[2]

Calculation of prognostic scores

Considering different model for end-stage liver disease (MELD) scores published, we used the MELD-Na score throughout this study.[16] The CLIF-C ACLF-D score was calculated according to Trebicka *et al.*: ((0.03 × age) + (0.45 × ascites) + (0.26 × ln(WBC)) – (0.37 × albumin) + (0.57 × ln(bilirubin)) + (1.72 × ln(creatinine)) + 3 × 10.[17]

MRI protocol

MRI exams were performed using a 3T MRI scanner (Magnetom Trio, A Tim System 3T; or Prisma[Fit], Siemens Healthcare, Erlangen, Germany). We injected a standard dose of GA (0.025 mmol/kg; Primovist in Europe and Eovist in the United States; Bayer Healthcare, Berlin, Germany) intravenously at a rate of 1.0 ml/s, immediately followed by a 20-ml saline flush. The contrast-enhanced sequence consisted of 3D, axial T1-weighted, volume-interpolated breath-hold examination sequences, obtained before and 20 min after contrast injection, as well as in the coronal view after injection of GA in the hepatobiliary phase. MRI protocol and acquisition parameters are given in Table S2 and have been described elsewhere.

Image analysis

We performed a qualitative or semi-quantitative and a quantitative analysis. For the semi-quantitative assessment, three radiologists (L.Be., A.B.-S., and N.B.) independently calculated the FLIS, derived from GA-enhanced MRI, on axial and coronal 20-minute hepatobiliary phase images[18] by the visual assessment of three features: (i) liver parenchymal enhancement or enhancement quality score, which compares liver to renal parenchymal enhancement. It assesses how well the hepatocytes take up the contrast agent; (ii) biliary contrast excretion or excretion quality score by rating the extent of filling of the bile ducts with contrast agent. It assesses how well the hepatocytes excrete the contrast agent; and (iii) portal vein sign, *i.e.* portal vein sign quality score, which compares the signal intensity of the portal vein to the signal intensity of the liver parenchyma. Each of these three features is scored on a scale of 0 to 2. The total FLIS score is the sum of the three individual scores, ranging from 0 (poor) to 6 (excellent).[19] The median of the three independent FLIS assessments/radiologists was used for the final analysis,[9] as previously described. The coronal T2-weighted 2D- and 3D-magnetic resonance cholangiopancreatography and T2-weighted HASTE images were reviewed for bile duct dilatation as a sign of mechanical cholestasis, and these patients were excluded.

For the quantitative analysis, two radiologists (S.P., with >8 years of experience in abdominal imaging, and a resident V.S., with 3 years of experience in abdominal imaging) independently extracted and calculated the following three quantitative parameters from the unenhanced images and the hepatobiliary phase of GA-enhanced MRI:

The RLE was calculated as follows: RLE = ($SI_{liver\ HBPenhanced}$ – $SI_{liver\ unenhanced}$)/($SI_{liver\ unenhanced}$)*100 (signal intensity, SI). The quantitative measurements came from four regions of interest (ROIs), drawn as large as possible, *i.e.* 2.0-5.0 cm^2, within homogeneous areas of the left lobe (segments II and III) and right lobe (segments VI and VIII).[20]

To measure the relative signal intensity of biliary contrast enhancement, ROIs ≤10 mm were placed in an optimal area of the upper end of the common bile duct (SI_{up}) and lower end of the common bile duct (SI_{low}) on an axial image. To reduce error, the SI of the ipsilateral erector spinae muscle (SI_{Muscle}) was measured using a similarly sized ROI. Finally, the relative enhancement ratio of the biliary system (REB) was calculated as follows: REB = (SI_{up}/SI_{muscle} + SI_{low}/SI_{muscle})/2 according to previous publications.[21]

Lastly, the liver-to-portal vein contrast ratio (LPC) was calculated as follows: ($SI_{liver\ enhanced}/SI_{portal\ vein\ enhanced}$).[22]

In addition, the longest diameter of the spleen was measured.

Statistical analyses

Statistical analyses were performed using IBM SPSS Statistics Version 28 (IBM, Armonk, NY), and R 4.5.2 (R Core Team, R Foundation for Statistical Computing, Vienna, Austria). Continuous variables were reported as mean (SD) for normally distributed data or median (IQR) if the distribution was skewed. Categorical variables were reported as number and percentage of patients with the specific characteristics.

Group comparisons were performed using the Mann-Whitney *U* test or an unpaired t-test for continuous variables, when applicable. Group comparisons of categorical variables were performed using the Fisher's exact test. Intra- and inter-observer variability were obtained using a mixed intraclass correlation coefficient model, with absolute agreements, single measures, and 95% CIs.

Follow-up was defined as the time between GA-MRI (*i.e.* baseline) and the events of interest, *i.e.* (i) development of ACLF or (ii) liver-related death,[23–25] or (iii) last clinical follow-up. Median time of follow-up was calculated by the reverse Kaplan-Meier method. Furthermore, uni- and multivariable competing risk regression analyses for development of ACLF/liver-related death were performed with liver transplantation as a competing event. Cumulative incidence functions were estimated using the Aalen–Johansen estimator, accounting for competing risks. Group differences were assessed using Gray's test. Time-dependent receiver-operating characteristic (ROC) curve analyses for ACLF/liver-related death (with liver transplantation as a competing risk) were calculated using the timeROC package in R. Cumulative incidence plots were used for visualisation of time-to-event analyses. Baseline characteristics which may have prognostic implications, as well as parameters which we considered of particular importance for the endpoint of interest, were included in the multivariable competing risk regression models. To evaluate whether FLIS and RLE/REB/LPC provide information that is independent from established prognostic indicators, we adjusted our models for either i) the CLIF-C ACLF-D score or ii) the MELD-Na score and albumin, while also accounting for removal or suppression of the primary aetiological factor as a time-dependent co-variable. Removal or suppression of the primary aetiological factor was defined by the initiation of antiviral therapy or reported alcohol abstinence.

A *p* value <0.05 was considered statistically significant.

Ethics

The retrospective evaluation of all consecutive MRI exams performed between 2011 and 2019 and associated clinical information was approved by the local Institutional Review Board, which waived the requirement for informed consent.

Results

Patient characteristics

We included 210 patients (n = 141 male; 67.2%) with a mean age of 54.4 ± 13.8 years (Fig. S1). The most common liver disease aetiologies were alcohol-related liver disease (n = 54; 25.7%) and viral hepatitis (hepatitis C virus: n = 61; 29%, hepatitis B virus: n = 14; 6.7%). The most common indication for MRI was the evaluation of liver nodules (n = 167; 79.5%). Patient characteristics according to the presence or absence of AD are provided in Table 1.

Inter-observer variability of semi-quantitative and quantitative GA-MRI imaging biomarkers

The inter-observer variability was good to perfect for all evaluated parameters without profound differences between most of the parameters (Table S3).

Semi-quantitative (FLIS) and quantitative scores change with clinical stage

There were significant differences in the median FLIS, RLE, LPC, and REB between patients with AD and clinically stable ACLD, as well as between patients with compensated ACLD and those with decompensated cirrhosis (*p* <0.001; Table 2).

Description of clinical outcomes

Overall, 49 patients (23.3%) developed ACLF, while 48 patients (22.9%) experienced liver-related death during a median follow-up of 34.6 months (95% CI 30.1–42.1). Detailed information on clinical outcomes according to disease stage can be found in Table S4.

Time-dependent AUROC analysis to predict ACLF/liver-related death

We evaluated the time-dependent predictive performance of MRI parameters for ACLF/liver-related death (Fig. 1). In patients with AD (Fig. 1A), FLIS showed the highest AUROC shortly after MRI, followed by a gradual decline over time, while remaining approximately 0.7 during the first 12 months. Similar patterns were observed in clinically stable ACLD, decompensated cirrhosis, and cACLD (Fig. 1B–D).

Low FLIS is a risk factor for ACLF development/liver-related death in AD

In AD (n = 49), 37 patients (76%) developed the composite endpoint of ACLF/liver-related death during a median estimated follow-up time of 8.0 months (95% CI 3.1-31.3).

Notably, patients experiencing the outcome of interest within their initial hospital stay (n = 8), compared with those who experienced the outcome after discharge (n = 29), had a significantly lower FLIS (1.1 ± 0.6 *vs.* 2.6 ± 1.9 points, *p* = 0.042).

Table 2. Comparison of scores according to disease stage.

Imaging characteristics, mean (SD)	AD, n = 49	Clinically stable ACLD, n = 161	Decompensated cirrhosis, n = 100	Compensated ACLD, n = 110
FLIS*	2.6 (1.9)	4.8 (1.6)	5.0 (1.4)	3.5 (2.1)
RLE*	54.8 (32.5)	93.3 (48.2)	97.8 (46.7)	69.9 (44.9)
REB*	1.9 (1.7)	8.9 (34.3)	9.6 (41.2)	4.6 (6.7)
LPC*	1.4 (0.5)	1.7 (0.6)	1.6 (0.5)	1.6 (0.7)

Group comparisons were performed using the Mann–Whitney *U* test or unpaired t test, as appropriate.
AD, acute decompensation; cACLD, compensated advanced chronic liver disease; FLIS, functional liver imaging score; LPC, liver to portal vein ratio; REB, relative enhancement radio of the biliary systems; RLE, relative liver enhancement.
*p value <0.001 when comparing AD with clinically stable ACLD as well as decompensated cirrhosis with cACLD.

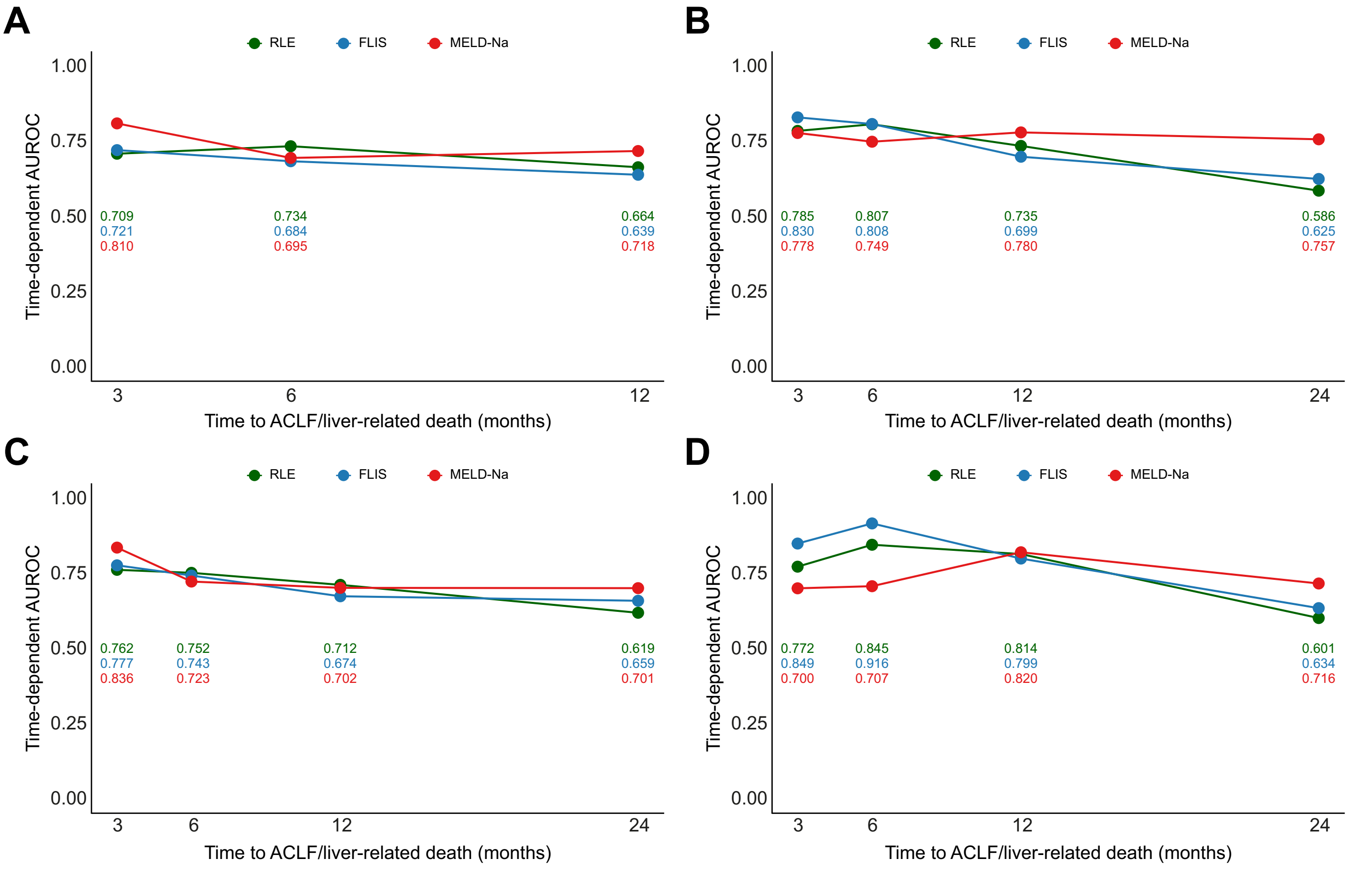

Fig. 1. Panels showing the time-dependent AUROC for prediction of ACLF/liver-related death. Panels showing the time-dependent AUROC for prediction of ACLF/liver-related death in (A) acute decompensation, (B) clinically stable advanced chronic liver disease, (C) decompensated cirrhosis, and (D) compensated advanced chronic liver disease stratified according to their FLIS. Time-dependent AUROCs were estimated using competing-risk time-dependent receiver operating characteristic analysis (timeROC package in R), with liver transplantation treated as a competing event. ACLF, acute-on-chronic liver failure; FLIS, functional liver imaging score; MELD, model for end-stage liver disease; RLE, relative liver enhancement.

In univariable competing-risk analyses, a low FLIS (0–3 points), corresponding to the previously established cut-off for poor liver function,[10] was identified as a risk factor for ACLF/liver-related death in patients with AD (Table 3) (subdistribution hazard ratio [SHR] 2.05, 95% CI 1.08–3.89, *p* = 0.028). Notably, neither the LPC nor the RLE were associated with the outcome of interest.

In multivariable analyses adjusted for MELD-Na score, aetiological cure, and albumin, a low FLIS remained an independent risk factor for ACLF/liver-related death (adjusted SHR [aSHR] 2.37, 95% CI 1.08-5.17, *p* = 0.031), while the association between low FLIS and ACLF/liver-related death did not attain statistical significance after accounting for CLIF-C ACLF-D instead of MELD-Na score and albumin. Importantly,

Table 3. Uni- and multivariable competing risk regression analyses of factors associated with ACLF/liver-related death during follow-up in AD (n = 49), considering liver transplantation as competing event.

	Univariable analysis			Multivariable analysis including FLIS & CLIF-C ACLF-D			Multivariable analysis including FLIS & MELD/albumin		
**Patient characteristics**	**SHR**	**95% CI**	***p* value**	**aSHR**	**95% CI**	***p* value**	**aSHR**	**95% CI**	***p* value**
MELD-Na, per point	1.11	1.05-1.17	**<0.001**	—	—	—	1.11	1.05-1.17	**<0.001**
CLIF-C ACLF-D	1.77	1.33-2.36	**<0.001**	1.68	1.26-2.25	**<0.001**	—	—	—
Albumin, per G/L	0.93	0.87-0.99	**0.047**	—	—	—	0.98	0.93-1.03	0.500
Spleen size, cm	0.95	0.87-1.04	0.270	—	—	—	—	—	—
RLE, %, per 10	0.90	0.80-1.02	0.090	—	—	—	—	—	—
LPC, per point	0.98	0.56-1.72	0.950	—	—	—	—	—	—
REB, per point*	0.02	0.01-0.38	**0.001**	—	—	—	—	—	—
FLIS, per point*	0.81	0.70-0.94	**0.006**	—	—	—	—	—	—
FLIS, 0-3 points	2.05	1.08-3.89	**0.028**	1.87	0.83-4.25	0.130	2.37	1.08-5.17	**0.031**
Aetiological cure	0.36	0.14-0.89	**0.027**	0.34	0.13-0.90	**0.030**	0.36	0.15-0.85	**0.020**

Subdistribution hazard ratios were estimated using Fine–Gray competing-risk regression, considering liver transplantation as a competing event.
Bold type indicates statistically significant values (*p* <0.05). AD, acute decompensation; ACLF, acute-on-chronic liver failure; aSHR, adjusted subdistribution hazard ratio; CLIF-C ACLF-D, Chronic Liver Failure Consortium acute-on-chronic liver failure development score; FLIS, functional liver imaging score; LPC, liver to portal vein contrast ratio; MELD, model for end-stage liver disease; REB, relative enhancement ratio of the biliary system; RLE, relative liver enhancement.
*The association with the outcome of interest did not attain statistical significance in multivariable analysis (data not shown).

the association between low FLIS and the outcome was dependent on the adjustment model, remaining significant after adjustment for MELD-Na and albumin but not after inclusion of the CLIF-C ACLF-D score.

Low FLIS may be a risk factor for ACLF/liver-related death in clinically stable ACLD, decompensated cirrhosis, or cACLD

One hundred sixty-one (76.7%) patients with clinically stable ACLD were included. Of these, 47 (29.2%) developed ACLF/liver-related death. Among 100 (47.6%)/110 (52.4%) patients with decompensated cirrhosis/cACLD, 60 (60%)/24 (21.8%) developed the outcome of interest.

FLIS was associated with ACLF/liver-related death in univariable analyses in clinically stable ACLD (SHR 2.60, 95% CI 1.39-4.85, *p* = 0.003; Fig. 2B, Table 5), decompensated cirrhosis (SHR 2.13, 95% CI 1.29-3.51, *p* = 0.003; Fig. 2C, Table 4), and cACLD (SHR 3.80, 95% CI 1.54-9.43, *p* = 0.004; Fig. 2D, Table 6). After adjusting for established prognostic indicators and aetiological cure, these associations did not remain statistically significant, except in cACLD (aSHR 2.76, 95% CI 1.02-7.44, *p* = 0.045). Overall, the strength and statistical significance of the association between FLIS and the outcome were attenuated after multivariable adjustment across disease stages. RLE/REB were in part associated with the outcome of interest in univariable analysis, but not in multivariable analyses.

Low FLIS and specific organ dysfunctions/failures

Finally, low FLIS was associated with liver dysfunction/failure among patients who developed ACLF during follow-up (*p* <0.001). Other organ dysfunction/failures, as well as minimum and maximum ACLF grades, were comparable (Table S5).

Discussion

ACLF is a highly dynamic syndrome that can occur in AD or decompensated cirrhosis, leading to multiple organ failure and high short-term mortality. Using competing risk regression analysis, we found that the FLIS was the only independent imaging biomarker for the development of ACLF/liver-related death in AD (*i.e.* the main at-risk population) and cACLD, and it was also associated with the outcome of interest in clinically stable ACLD and decompensated cirrhosis. This reflects the strength of FLIS as a versatile prognostic imaging biomarker with independent prognostic abilities.

However, due to stage-specific/clinical context-dependent analyses (*i.e.* a strength of our study), FLIS did not attain statistical significance in some multivariable models. Notably, the prognostic impact of low FLIS was dependent on the adjustment model, with attenuation of the effect after inclusion of established prognostic scores, particularly CLIF-C ACLF-D. This likely reflects partial overlap between FLIS and these scores, as both capture key aspects of hepatic dysfunction and systemic disease severity. This may be attributed to the limited number of events and the

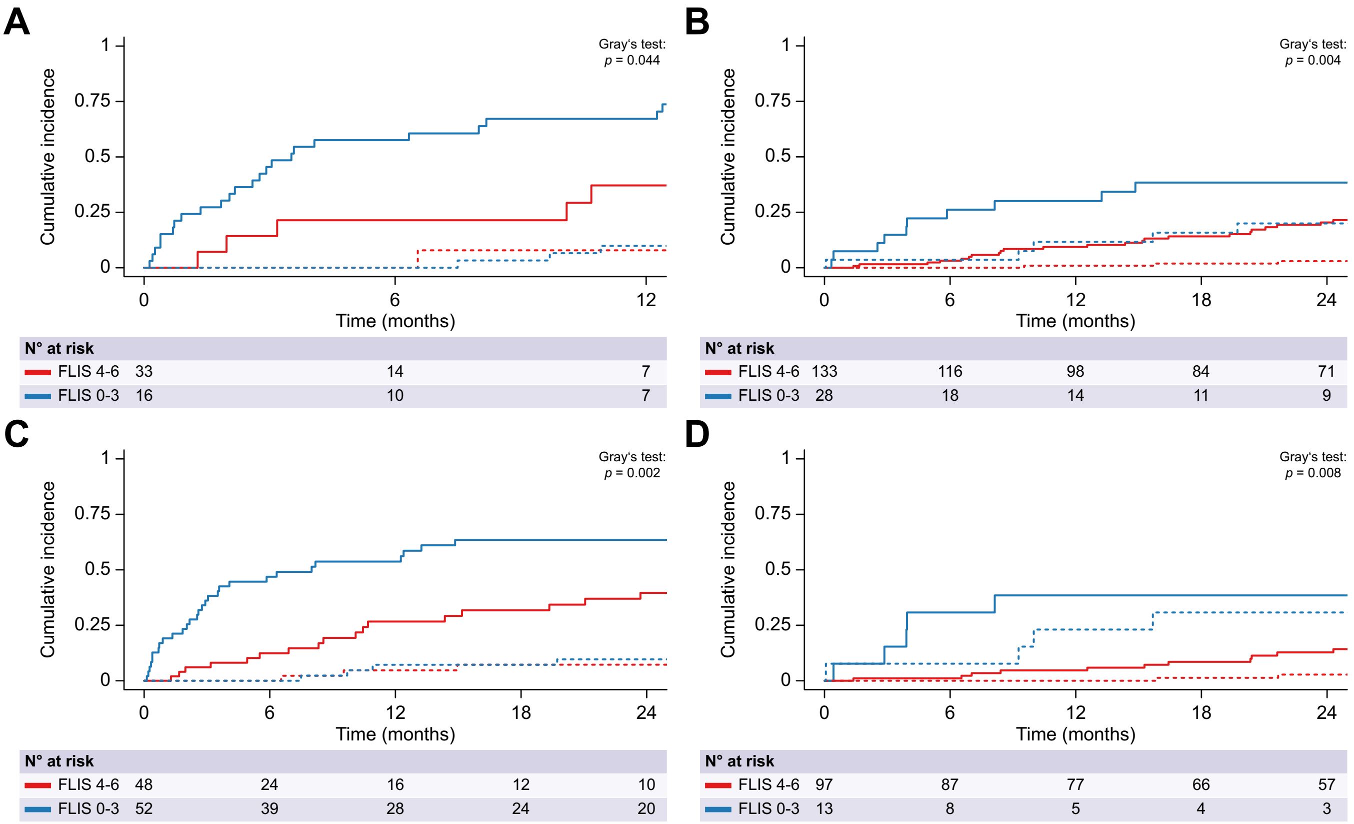

Fig. 2. Cumulative incidence curve for the development of ACLF/liver-related death. Cumulative incidence curve for the development of ACLF/liver-related death in (A) acute decompensation, (B) clinically stable advanced chronic liver disease, (C) decompensated cirrhosis, and (D) compensated advanced chronic liver disease stratified according to their FLIS. The dotted lines denote the respective incidence of the competing risk (liver transplantation). ACLF, acute-on-chronic liver failure; FLIS, functional liver imaging score. Group differences were assessed using Gray's test, accounting for liver transplantation as a competing event.

Table 4. Uni- and multivariable competing risk regression analyses of factors associated with ACLF/liver-related death during follow-up in decompensated cirrhosis (n = 100), considering liver transplantation as a competing event.

	Univariable analysis			Multivariable analysis including FLIS & CLIF-C ACLF-D			Multivariable analysis including FLIS & MELD/albumin		
**Patient characteristics**	**SHR**	**95% CI**	***p* value**	**aSHR**	**95% CI**	***p* value**	**aSHR**	**95% CI**	***p* value**
MELD-Na, per point	1.12	1.07-1.17	**<0.001**	—	—	—	1.09	1.04-1.15	**<0.001**
CLIF-C ACLF-D	1.84	1.46-2.32	**<0.001**	1.75	1.40-2.19	**<0.001**	—	—	—
Albumin, per G/L	0.93	0.88-0.98	**0.004**	—	—	—	0.97	0.92-1.02	0.180
Spleen size, cm	0.93	0.86-1.02	0.110	—	—	—	—	—	—
RLE, %, per 10	0.91	0.85-0.98	**0.011**	—	—	—	—	—	—
LPC, per point	0.92	0.54-1.55	0.740	—	—	—	—	—	—
REB, per point, per 10	0.53	0.22-1.25	0.150	—	—	—	—	—	—
FLIS, per point*	0.82	0.73-0.92	**<0.001**	—	—	—	—	—	—
FLIS, 0-3 points	2.13	1.29-3.51	**0.003**	1.68	0.94-3.00	0.082	1.50	0.83-2.68	0.180
Aetiological cure	0.42	0.24-0.73	**0.002**	0.43	0.24-0.77	**0.004**	0.47	0.26-0.84	**0.010**

Subdistribution hazard ratios were estimated using Fine–Gray competing-risk regression, considering liver transplantation as a competing event.
Bold type indicates statistically significant values (p <0.05). ACLF, acute-on-chronic liver failure; aSHR, adjusted subdistribution hazard ratio; CLIF-C ACLF-D, Chronic Liver Failure consortium acute-on-chronic liver failure score; FLIS, functional liver imaging score; LPC, liver to portal vein contrast ratio; MELD, model for end-stage liver disease; REB, relative enhancement ratio of the biliary system; RLE, relative liver enhancement.
*The association with the outcome of interest did not attain statistical significance in multivariable analysis (data not shown).

Table 5. Uni- and multivariable competing risk regression analyses of factors associated with ACLF/liver-related death during follow-up in clinically stable ACLD (n = 161), considering liver transplantation as competing event.

	Univariable analysis			Multivariable analysis including FLIS & CLIF-C ACLF-D			Multivariable analysis including FLIS & MELD/albumin		
**Patient characteristics**	**SHR**	**95% CI**	***p* value**	**aSHR**	**95% CI**	***p* value**	**aSHR**	**95% CI**	***p* value**
MELD-Na, per point	1.10	1.04-1.16	**<0.001**	—	—	—	1.06	0.99-1.13	0.110
Aetiological cure	0.88	0.50-1.55	0.650	0.88	0.46-1.66	0.680	0.74	0.40-1.36	0.330
CLIF-C ACLF-D	1.79	1.33-2.40	**<0.001**	1.75	1.30-2.35	**<0.001**	—	—	—
Albumin, per G/L	0.91	0.86-0.96	**<0.001**	—	—	—	0.95	0.88-1.01	0.110
Spleen size, cm	1.05	0.98-1.11	0.160	—	—	—	—	—	—
RLE, %, per 10	0.92	0.85-0.99	**0.020**	—	—	—	—	—	—
LPC, per point	0.96	0.48-1.90	0.900	—	—	—	—	—	—
REB, per point, per 10	1.02	1.00-1.04	**0.046**	—	—	—	—	—	—
FLIS, per point*	0.79	0.66-0.93	**0.006**	—	—	—	—	—	—
FLIS, 0-3 points	2.60	1.39-4.85	**0.003**	1.47	0.65-3.36	0.350	1.58	0.73-3.45	0.250

Subdistribution hazard ratios were estimated using Fine–Gray competing-risk regression, considering liver transplantation as a competing event.
Bold type indicates statistically significant values (p <0.05). ACLD, advanced chronic liver disease; ACLF, acute-on-chronic liver failure; aSHR, adjusted subdistribution hazard ratio; CLIF-C ACLF-D, Chronic Liver Failure Consortium acute-on-chronic liver failure development score; FLIS, functional liver imaging score; LPC, liver to portal vein contrast ratio; MELD, model for end-stage liver disease; REB, relative enhancement ratio of the biliary system; RLE, relative liver enhancement.
*The association with the outcome of interest did not attain statistical significance in multivariable analysis (data not shown).

Table 6. Uni- and multivariable competing risk regression analyses of factors associated with ACLF/liver-related death during follow-up in cACLD (n = 110), considering liver transplantation as competing event.

	Univariable analysis			Multivariable analysis including FLIS & CLIF-C ACLF-D			Multivariable analysis including FLIS & MELD/albumin		
**Patient characteristics**	**SHR**	**95% CI**	***p* value**	**aSHR**	**95% CI**	***p* value**	**aSHR**	**95% CI**	***p* value**
MELD-Na, per point	1.09	1.01-1.17	**0.019**	—	—	—	1.01	0.93-1.11	0.760
Aetiological cure	0.66	0.27-1.61	0.360	1.02	0.32-3.23	0.970	0.62	0.23-1.67	0.340
CLIF-C ACLF-D	1.85	1.15-2.97	**0.011**	1.77	1.08-2.91	**0.024**	—	—	—
Albumin, per G/L	0.88	0.81-0.96	**0.005**	—	—	—	0.90	0.81-0.99	**0.031**
Spleen size, cm	1.06	0.98-1.15	0.140	—	—	—	—	—	—
RLE, %, per 10	0.93	0.82-1.06	0.270	—	—	—	—	—	—
LPC, per point	0.65	0.25-1.74	0.400	—	—	—	—	—	—
REB, per point, per 10	1.03	1.01-1.05	**0.003**	—	—	—	—	—	—
FLIS, per point*	0.71	0.53-0.95	**0.023**	—	—	—	—	—	—
FLIS, 0-3 points	3.80	1.54-9.43	**0.004**	1.73	0.33-9.07	0.520	2.76	1.02-7.44	**0.045**

Subdistribution hazard ratios were estimated using Fine–Gray competing-risk regression, considering liver transplantation as a competing event.
Bold type indicates statistically significant values (p <0.05). ACLF, acute-on-chronic liver failure; aSHR, adjusted subdistribution hazard ratio; cACLD, compensated advanced chronic liver disease; CLIF-C ACLF-D, Chronic Liver Failure consortium acute-on-chronic liver failure score; FLIS, functional liver imaging score; LPC, liver to portal vein contrast ratio; MELD, model for end-stage liver disease; REB, relative enhancement ratio of the biliary system; RLE, relative liver enhancement.
*The association with the outcome of interest did not attain statistical significance in multivariable analysis (data not shown).

consideration of removal or suppression of the primary aetiological factor, *i.e*. a key predictive factor not known at baseline.

Importantly, FLIS was independently predictive of ACLF/liver-related death in AD after accounting for MELD-Na and albumin. Despite being independently linked to ACLF/liver-related death in cACLD, the added value of FLIS in less severe clinical contexts (*i.e*. outside AD) is questionable, as indicated by the lack of an association with ACLF/liver-related death among patients with clinically stable ACLD.

Importantly, the persistence of statistical significance in AD after adjustment for MELD-Na and albumin suggests that FLIS may provide incremental prognostic information particularly in this high-risk and dynamic population.

Notably, FLIS showed time-dependent decreases in the AUROC to predict ACLF/liver-related death, with a high AUROC within 6 months among patients with AD and clinically stable ACLD. The earlier decline in AUROCs may reflect its more dynamic natural history compared to clinically stable ACLD, including a potential variation in FLIS over time. Updating FLIS over time may increase its prognostic utility. This concept has already been shown for liver stiffness measurement studied in another clinical context by our group[26] and others.[27]

Due to its high short-term mortality and restricted treatment possibilities, the ideal strategy to reduce the burden of life-threatening ACLF would be its prevention. For this, risk stratification tools for patients with ACLD are needed. Recently, the CLIF-C ACLF-D score, a blood-based model combining biomarkers and clinical information, was developed to predict ACLF in patients with AD.[15] Larger samples of patients with AD are required to establish that FLIS adds to the information provided by the CLIF-C ACLF-D score, *i.e.* a model that was specifically built for this purpose, includes clinical information that is not entirely objective (ascites), and requires sophisticated calculations.

While portal hypertension is the main disease-driving mechanism for hepatic decompensation in ACLD, the main hypothesis for ACLF development is intense systemic inflammation that triggers (multi-)organ dysfunction and ultimately failure.[28] In line with this, C-reactive protein,[29] as a marker of systemic inflammation, has been shown to be an important predictor of ACLF development in patients with AD. Next to immunometabolic changes in white blood cells, it seems that severe mitochondrial dysfunction governs inflammatory responses in patients with AD and ACLF.[30] In agreement with this, sophisticated models that include metabolites reflecting systemic inflammation, mitochondrial dysfunction, and sympathetic system activation, might further improve risk stratification,[31] compared to the CLIF-C ACLF-D alone. The comparative performance of FLIS as a simple imaging biomarker and such tools may be evaluated in further studies. Notably, next to liver imaging-based scores, body composition and (imaging-based) sarcopenia assessments have been shown to predict ACLF and short-term mortality in patients with ACLF.[32]

We can only hypothesise on the pathomechanisms underlying the predictive utility of FLIS for disease progression (*i.e*. ACLF). Since indocyanine green clearance and GA uptake and excretion (*i.e.* FLIS) rely on comparable transporter mechanisms/functional capabilities,[33–35] results on indocyanine green retention after 15 min might help to better understand the link between FLIS and ACLF.[36] Beyond HVPG and MELD score, indocyanine green retention after 15 min was correlated with systemic inflammation, endothelial dysfunction, and enhanced liver fibrosis test (*i.e*. extracellular matrix remodelling) in both cACLD and decompensated cirrhosis, and markers of circulatory dysfunction in decompensated cirrhosis.[37,38] This indicates that hepatic uptake and excretion, if assessed by a functional test rather than standard laboratory markers, reflect several mechanisms that drive ACLF development, thereby providing a rationale for the observed prognostic utility of FLIS. In addition, low FLIS was primarily linked to the occurrence of liver dysfunction/failure, suggesting that functional impairment on an imaging level may precede liver failure, as assessed by CLIF-SOFA.

FLIS is a semi-quantitative radiological biomarker that can easily be assessed. It requires neither specific hardware nor software, nor calculations. Our findings regarding inter-reader reliability are consistent with the meta-analysis by Kim *et al.*, which reported a pooled inter-reader reliability of 0.93 for the FLIS.[39] We compared the predictive ability of the semi-quantitative FLIS to that of the three quantitative versions of its parameters (RLE, REB, and LPC). On multivariable analysis, the FLIS was the only imaging parameter that was an independent risk factor for the development of ACLF/liver-related death in AD and even cACLD. From a mechanistic perspective, the attenuation of the effect size after adjustment suggests that FLIS may reflect, at least in part, pathophysiological processes that are also captured by established prognostic scores. This is further supported by the more pronounced attenuation observed when adjusting for CLIF-C ACLF-D compared to MELD-based models, suggesting that incorporation of systemic inflammation and additional clinical parameters may reduce the independent contribution of FLIS.

Furthermore, we found that RLE, REB, and LPC have excellent reliability, shown by their very high intraclass correlation values. In addition, these quantitative MRI-derived parameters differed between clinically stable ACLD and AD. However, these indices failed to independently predict ACLF/liver-related death in the relevant clinical subgroups, in particular in AD.

To the best of our knowledge, this is the first manuscript to report on a liver imaging biomarker that can predict the development of ACLF/liver-related death in AD and clinically stable ACLD.[9] The FLIS, derived from GA-enhanced MRI, was conceived in 2016 to predict the transplant-free survival of patients after orthotopic LT.[19] Since then, its ability to evaluate liver function in chronic liver disease has been demonstrated.[40] While some risk stratification tools/markers have been explicitly developed for different disease aetiologies, multiple studies have shown that the FLIS is applicable to a wide range of ACLD.[10,41–43] Thus, it is an excellent candidate for an imaging biomarker.

Our study has several limitations. First, the retrospective design of our single-centre study could have led to a selection bias; however, the study allowed for a reasonably long clinical follow-up and a relatively high number of endpoints. Notably, rates of loss to follow-up and missed events were minimised. We thoroughly reviewed electronic health records of the Vienna hospital association and nation-wide electronic health records. Moreover, we also performed searches of the liver transplant database of our institution (*i.e*. the only transplant centre in

eastern Austria) and examined the nationwide death registry. Since complete information on (cause of) death is guaranteed by the latter measure, we included liver-related death in all composite endpoints to ensure the ascertainment of the most severe disease courses. Second, regarding the RLE, LPC, and REB, all measurements were made on a 3T MRI scanner, but the signal intensities may be dependent upon hardware and software. Third, the small ROIs, *i.e.* ≤1 cm, used to obtain signal intensities in the portal vein and common bile duct to obtain the LPC and REB, respectively, may make it difficult to reproduce these values. This may have contributed to their inability to predict ACLF, although the intraclass correlation coefficient was excellent for both parameters. Finally, our findings have yet to be validated externally. However, we have neither developed a novel model nor established a new FLIS cut-off, thereby decreasing the chance of overly optimistic results.

In conclusion, the FLIS is a simple prognostic imaging biomarker capable of predicting the composite endpoint of ACLF development and liver-related death in AD. Risk stratification using FLIS may help identify patients who could benefit from intensified monitoring or timely evaluation for liver transplantation.

Affiliations

[1]*Department of Biomedical Imaging and Image-Guided Therapy, Medical University of Vienna, Vienna, Austria;* [2]*Hepatic Hemodynamic Lab, Medical University of Vienna, Vienna, Austria;* [3]*Division of Gastroenterology and Hepatology, Department of Medicine III, Medical University of Vienna, Vienna, Austria;* [4]*Department of Diagnostic and Interventional Radiology, Clinic Donaustadt, Vienna Healthcare Group, Vienna, Austria;* [5]*Department of Internal Medicine IV, Clinic Ottakring, Vienna, Austria;* [6]*Computational Imaging Research Lab, Department of Biomedical Imaging and Image-guided Therapy, Medical University of Vienna, Vienna, Austria;* [7]*Christian Doppler Laboratory for Machine Learning Driven Precision Imaging, Department of Biomedical Imaging and Image-guided Therapy, Medical University of Vienna, Vienna, Austria*

Abbreviations

ACLD, advanced chronic liver disease; ACLF, acute-on-chronic liver failure; AD, acute decompensation; AUROC, area under the receiver-operating characteristic; aSHR, adjusted subdistribution hazard ratio; cACLD, compensated advanced chronic liver disease; CTP, Child-Turcotte-Pugh; FIB-4, fibrosis-4 score; FLIS, functional liver imaging score; GA, gadoxetic acid; HVPG, hepatic venous pressure gradient; LPC, liver to portal vein contrast ratio; MELD, model for end-stage liver disease; REB, relative enhancement ratio of the biliary system; RLE, relative liver enhancement; ROC, receiver operating characteristic; ROI, region of interest; SI, signal intensity.

Financial support

European Union's Horizon 2020 research and innovation programme under grant agreement No 101136299. This work has been partly funded by the European Union's Horizon Europe research and innovation programme under grant agreement No. 101136299 (ARTEMIS).

Conflicts of interest

L.Be. received speaker fees from Takeda, and Lilly. A.B.-S. received honoraria for lectures and a consultancy from Bayer, without relation to the present article. U.A. Siemens Healthineers Speaker Bureau, Bayer Consultancy, without relation to the present article. M.T. served as a speaker and/or consultant and/or advisory board member for Abbvie, Agomab, Albireo, BiomX, Falk, Boehringer Ingelheim, Bristol-Myers Squibb, Chemomab, Falk, Genfit, Gilead, Hightide, Intercept, Ipsen, Janssen, MSD, Novartis, Phenex, Pliant, Regulus, Siemens and Shire, and received travel support from AbbVie, Falk, Gilead, Intercept and Jannsen, as well as grants/research support from Albireo, Alnylam, Cymabay, Falk, Genentech, Gilead, Intercept, MSD, Takeda, and UltraGenyx. He is also co-inventor of patents on the medical use of 24-norursodeoxycholic acid. M.M. received grants from Echosens, served as a speaker and/or consultant and/or advisory board member for AbbVie, Collective Acumen, Echosens, Gilead, Ipsen, Takeda, and W. L. Gore & Associates, and received travel support from AbbVie and Gilead. T.R. received grant support from AbbVie, Boehringer-Ingelheim, Gilead, Intercept, MSD, Myr Pharmaceuticals, Philips Healthcare, Pliant, Siemens, and W. L. Gore & Associates; speaking honoraria from AbbVie, Gilead, W. L. Gore & Associates, Intercept, Roche, and MSD; consulting/advisory board fees from AbbVie, Bayer, Boehringer-Ingelheim, Gilead, Intercept, MSD, and Siemens; and travel support from AbbVie, Boehringer-Ingelheim, Gilead, and Roche. All other authors declare no relationships with any companies whose products or services may be related to the subject matter of the article.

Please refer to the accompanying ICMJE disclosure forms for further details.

Authors' contributions

N.B., L.Be., L.Ba, S.P.L, R.A., A.K., J.K., K.P., B.S., G.S., V.S., S.P., D.S., J.C.H., A.B., M.M., T.R., U.A., M.T. (acquisition of data and critical revision of manuscript); S.P.L, N.B., L.Be., L.Ba., A.B-S, M.M. (study concept and design, analysis, and interpretation of data, statistical analysis, drafting of manuscript); S.P.L., L.Ba., L.Be., N.B., A.B.-S., M.T., M.M., T.R., U.A. (study concept and design, interpretation of data, drafting of manuscript, critical revision of manuscript). All authors read and commented on the final manuscript.

Acknowledgement

We acknowledge the great support from the Department of IT-Systems & Communications of the Medical University of Vienna who provided clinical and laboratory data.

Supplementary data

Supplementary data to this article can be found online at https://doi.org/10.1016/j.jhepr.2026.101928.

References

Author names in bold designate shared co-first authorship

[1] Qiu B, Liang JX, Romero Gomez M. The efficacy and safety of granulocyte colony-stimulating factor in the treatment of acute-on-chronic liver failure: a systematic review and meta-analysis. PLoS One 2023;18:e0294818.

[2] Moreau R, Jalan R, Gines P, et al. Acute-on-chronic liver failure is a distinct syndrome that develops in patients with acute decompensation of cirrhosis. Gastroenterology 2013;144:1426–1437. 1437.e1421-1429.

[3] Kumar R, Mehta G, Jalan R. Acute-on-chronic liver failure. Clin Med (Lond) 2020;20:501–504.

[4] European Association for the Study of the Liver. EASL Clinical Practice Guidelines on acute-on-chronic liver failure. J Hepatol 2023;79:461–491.

[5] Pose E, Napoleone L, Amin A, et al. Safety of two different doses of simvastatin plus rifaximin in decompensated cirrhosis (LIVERHOPE-SAFETY): a randomised, double-blind, placebo-controlled, phase 2 trial. The Lancet Gastroenterol Hepatol 2020;5:31–41.

[6] Juanola A, Ma AT, de Wit K, et al. Novel prognostic biomarkers in decompensated cirrhosis: a systematic review and meta-analysis. Gut 2023;73:156–165.

[7] European Association for the Study of the Liver. EASL Clinical Practice Guidelines on non-invasive tests for evaluation of liver disease severity and prognosis - 2021 update. J Hepatol 2021;75:659–689.

[8] Gustot T, Fernandez J, Garcia E, et al. Clinical Course of acute-on-chronic liver failure syndrome and effects on prognosis. Hepatology 2015;62:243–252.

[9] **Bastati N**, **Beer L**, Ba-Ssalamah A, et al. Gadoxetic acid-enhanced MRI-derived functional liver imaging score (FLIS) and spleen diameter predict outcomes in ACLD. J Hepatol 2022;77:1005–1013.

[10] **Bastati N**, **Beer L**, Mandorfer M, et al. Does the functional liver imaging score derived from gadoxetic acid-enhanced MRI predict outcomes in chronic liver disease? Radiology 2020;294:98–107.

[11] **Haider L**, **Mandorfer M**, Gungoren Z, et al. Noninvasive monitoring of liver disease regression after hepatitis C eradication using gadoxetic acid-enhanced MRI. Contrast Media Mol Imaging 2018;2018:8489709.

[12] Sterling RK, Lissen E, Clumeck N, et al. Development of a simple noninvasive index to predict significant fibrosis in patients with HIV/HCV coinfection. Hepatology 2006;43:1317–1325.
[13] de Franchis R, Bosch J, Garcia-Tsao G, et al. Baveno VII - renewing consensus in portal hypertension. J Hepatol 2022;76:959–974.
[14] **Semmler G**, **Hartl L**, Mendoza YP, et al. Simple blood tests to diagnose compensated advanced chronic liver disease and stratify the risk of clinically significant portal hypertension. Hepatology 2024;80:887–900.
[15] Trebicka J, Fernandez J, Papp M, et al. The PREDICT study uncovers three clinical courses of acutely decompensated cirrhosis that have distinct pathophysiology. J Hepatol 2020;73:842–854.
[16] Kim WR, Biggins SW, Kremers WK, et al. Hyponatremia and mortality among patients on the liver-transplant waiting list. New Engl J Med 2008;359:1018–1026.
[17] Trebicka J, Fernandez J, Papp M, et al. The PREDICT study uncovers three clinical courses of acutely decompensated cirrhosis that have distinct pathophysiology. J Hepatol 2020;73:842–854.
[18] Van Beers BE, Pastor CM, Hussain HK. Primovist, Eovist: what to expect? J Hepatol 2012;57:421–429.
[19] Bastati N, Wibmer A, Tamandl D, et al. Assessment of orthotopic liver transplant graft survival on gadoxetic acid-enhanced magnetic resonance imaging using qualitative and quantitative parameters. Invest Radiol 2016;51(11):728–734.
[20] Beer L, Mandorfer M, Bastati N, et al. Inter-and intra-reader agreement for gadoxetic acid–enhanced MRI parameter readings in patients with chronic liver diseases. Eur Radiol 2019;29:6600–6610.
[21] Liu C, Sun Y, Yang Y, et al. Gadobenate dimeglumine–enhanced biliary imaging from the hepatobiliary phase can predict progression in patients with liver cirrhosis. Eur Radiol 2021;31:5840–5850.
[22] Zhang W, Wang X, Miao Y, et al. Liver function correlates with liver-to-portal vein contrast ratio during the hepatobiliary phase with Gd-EOB-DTPA-enhanced MR at 3 Tesla. Abdom Radiol 2018;43:2262–2269.
[23] Ripoll C, Groszmann R, Garcia-Tsao G, et al. Hepatic venous pressure gradient predicts clinical decompensation in patients with compensated cirrhosis. Gastroenterology 2007;133:481–488.
[24] Scheiner B, Steininger L, Semmler G, et al. Controlled attenuation parameter does not predict hepatic decompensation in patients with advanced chronic liver disease. Liver Int 2019;39:127–135.
[25] Mandorfer M, Kozbial K, Schwabl P, et al. Changes in HVPG predict hepatic decompensation in patients who achieved SVR to IFN-free therapy. Hepatology 2020;71(3):1023–1036. Baltimore, Md.
[26] Semmler G, Yang Z, Fritz L, et al. Dynamics in liver stiffness measurements predict outcomes in advanced chronic liver disease. Gastroenterology 2023;165:1041–1052.
[27] Wong YJ, Chen VL, Abdulhamid A, et al. Comparing serial and current liver stiffness measurements to predict decompensation in compensated advanced chronic liver disease patients. Hepatology 2025;81:523–531.
[28] Clària J, Arroyo V, Moreau R. Roles of systemic inflammatory and metabolic responses in the pathophysiology of acute-on-chronic liver failure. JHEP Rep : innovation Hepatol 2023;5:100807.
[29] Zanetto A, Pelizzaro F, Campello E, et al. Severity of systemic inflammation is the main predictor of ACLF and bleeding in individuals with acutely decompensated cirrhosis. J Hepatol 2023;78:301–311.
[30] Zhang IW, Curto A, López-Vicario C, et al. Mitochondrial dysfunction governs immunometabolism in leukocytes of patients with acute-on-chronic liver failure. J Hepatol 2022;76:93–106.
[31] Weiss E, de la Peña-Ramirez C, Aguilar F, et al. Sympathetic nervous activation, mitochondrial dysfunction and outcome in acutely decompensated cirrhosis: the metabolomic prognostic models (CLIF-C MET). Gut 2023;72:1581–1591.
[32] Mangana Del Rio T, Sacleux SC, Vionnet J, et al. Body composition and short-term mortality in patients critically ill with acute-on-chronic liver failure. JHEP Rep 2023;5:100758.
[33] Haegele S, Reiter S, Wanek D, et al. Perioperative non-invasive indocyanine green-clearance testing to predict postoperative outcome after liver resection. PLoS One 2016;11:e0165481.
[34] Le Vee M, Lecureur V, Stieger B, et al. Regulation of drug transporter expression in human hepatocytes exposed to the proinflammatory cytokines tumor necrosis factor-alpha or interleukin-6. Drug Metab Dispos 2009;37:685–693.
[35] Møller S, la Cour Sibbesen E, Madsen JL, Bendtsen F. Indocyanine green retention test in cirrhosis and portal hypertension: accuracy and relation to severity of disease. J Gastroenterol Hepatol 2019;34:1093–1099.
[36] Jachs M, Carvalho T, Simbrunner B, et al. Indocyanine green clearance correlates with biomarkers reflecting key disease-driving mechanisms in advanced chronic liver disease and predicts acute-on-chronic liver failure and death. Aliment Pharmacol Ther 2026;63(6):874–884.
[37] De Gasperi A, Mazza E, Prosperi M. Indocyanine green kinetics to assess liver function: ready for a clinical dynamic assessment in major liver surgery? World J Hepatol 2016;8:355–367.
[38] Jachs MHL, Simbrunner B, Balcar L, et al. Indocyanine green clearance reflects various pathophysiological mechanisms and independently predicts liver-related events in ACLD. J Hepatol 2023;78:S286.
[39] Kim NH, Kang JH. Inter-reader reliability of functional liver imaging score derived from gadoxetic acid-enhanced MRI: a meta-analysis. Abdom Radiol (NY) 2023;48:886–894.
[40] Poetter-Lang S, Bastati N, Messner A, et al. Quantification of liver function using gadoxetic acid-enhanced MRI. Abdom Radiol (NY) 2020;45:3532–3544.
[41] Tang G, Liu J, Liu P, et al. Evaluation of liver function in patients with liver cirrhosis and chronic liver disease using functional liver imaging scores at different acquisition time points. Front Genet 2022;13:1071025.
[42] Lee HJ, Hong SB, Lee NK, et al. Validation of functional liver imaging scores (FLIS) derived from gadoxetic acid-enhanced MRI in patients with chronic liver disease and liver cirrhosis: the relationship between Child-Pugh score and FLIS. Eur Radiol 2021;31:8606–8614.
[43] Aslan S, Eryuruk U, Tasdemir MN, et al. Determining the efficacy of functional liver imaging score (FLIS) obtained from gadoxetic acid-enhanced MRI in patients with chronic liver disease and liver cirrhosis: the relationship between Albumin-Bilirubin (ALBI) grade and FLIS. Abdom Radiol (NY) 2022;47:2325–2334.

Keywords: Magnetic resonance imaging; Liver disease; Cirrhosis; ACLF.
Received 15 January 2025; received in revised form 6 June 2026; accepted 11 June 2026; Available online 17 June 2026

Journal of Hepatology, Volume *8*

Supplemental information

Functional liver imaging score (FLIS): A prognostic biomarker for acute-on-chronic liver failure and liver-related mortality

Sarah Poetter-Lang, Lorenz Balcar, Ahmed Ba-Ssalamah, Nina Bastati, Raphael Ambros, Antonia Kristic, Julia Krawanja, Katharina Pomej, Benedikt Simbrunner, Georg Semmler, Victor Schmidbauer, Svitlana Pochepnia, Daniel Sobotka, Jacqueline C. Hodge, Ulrike Attenberger, Michael Trauner, Thomas Reiberger, Lucian Beer, and Mattias Mandorfer

Functional liver imaging score (FLIS): A prognostic biomarker for acute-on-chronic liver failure and liver-related mortality

Sarah Poetter-Lang, Lorenz Balcar, Ahmed Ba-Ssalamah, Nina Bastati, Raphael Ambros, Antonia Kristic, Julia Krawanja, Katharina Pomej, Benedikt Simbrunner, Georg Semmler, Viktor Schmidbauer, Svitlana Pochepnia, Daniel Sobotka, Jacqueline C. Hodge, Ulrike Attenberger, Michael Trauner, Thomas Reiberger, Lucian Beer, Mattias Mandorfer

Table of contents

Table S1……………………………………………………………………………2

Table S2……………………………………………………………………………3

Table S3……………………………………………………………………………4

Table S4……………………………………………………………………………5

Table S1……………………………………………………………………………6

Fig. S1…………………………………………………………………..…..7

Table S1. Comparison of imaging characteristics of patients with vs without ACLF at study inclusion.

Imaging characteristics, mean (SD)	No ACLF at baseline, n=210	ACLF at baseline, n=6
FLIS	4.3 (1.9)	1.1 (3.2)
RLE	84.6 (47.8)	56.1 (25.2)
REB	7.2 (30.2)	1.1 (0.7)
LPC	1.6 (0.6)	1.2 (0.3)
ACLF, acute-on-chronic liver failure; FLIS, functional liver imaging score; LPC, liver to portal vein ratio; REB, relative enhancement ratio of the biliary system; RLE, relative liver enhancement; SD, standard deviation		

Table S2. MRI parameters for 3T images.

Sequence	Section Thickness (mm)	TR (msec)	TE (msec)	FOV (mm)	Phase Direction	Flip Angle
GRE-T1 (flash 2D) in-phase	5	130	2.46	350	AP	70
GRE-T1 (flash 2D) opposed-phase	5	131	3.69	350	AP	70
T1 VIBE SPAIR axial	1.7	2.67	0.97	430	AP	13
T1 VIBE SPAIR coronal	2	2.6	0.92	500	RL	13
T2 Haste coronal	4.5	805	76	450	RL	141
DWI axial TSE-EP	6	1700	73	380	AP	---
T2 MRCP 2D-HASTE	45	5500	454	380	RL	180
T2 MRCP 3D-SPACE	0.9	2400	707	350	RL	variable
T2 Haste axial fs.	5	1800	150	400	AP	150

2D, two-dimensional; FOV, field of view; fs, fat saturation; GRE, gradient echo; HASTE, half-fourier-acquired single-shot turbo spin echo; MRCP, magnetic resonance cholangiopancreatography; MRI, magnetic resonance imaging; SPAIR, spectral attenuated inversion recovery; TE, echo time; TR, repetition time; TSE, turbo spin echo; VIBE, volumetric interpolated breath-hold examination

Table S3. Inter-observer variability of semi-quantitative and quantitative gadoxetic acid scores.

**Variable**	**ICC**	**95%CI**
RLE	0.95	0.88-0.98
LPC	0.91	0.88-0.93
REB	0.82	0.75-0.87
FLIS	0.90	0.82-0.94

CI, confidence interval; FLIS, functional liver imaging score; ICC, intraclass correlation coefficient; LPC, liver to portal vein ratio; REB relative enhancement ratio of the biliary system; RLE, relative liver enhancement

Table S4. Summary of clinical outcomes during follow-up.

	AD, n=49	Clinically stable ACLD, n=161	Decompensated cirrhosis, n=100	Compensated ACLD, n=110
Acute-on-chronic liver failure, n (%)	20 (41%)	29 (18%)	33 (33%)	16 (15%)
Liver transplantation, n (%)	5 (10%)	11 (7%)	8 (8%)	8 (7%)
Deaths, n (%)	25 (51%)	23 (14%)	37 (37%)	11 (10%)
Liver-related deaths, n (%)	23 (47%)	22 (14%)	34 (34%)	11 (10%)
Acute-on-chronic liver failure or liver-related death, n (%)	37 (76%)	47 (29%)	60 (60%)	24 (22%)
Median time to acute-on-chronic liver failure/liver-related death; months (95%CI)	8.0 (3.1-31.3)	34.6 (30.1-41.3)	32.7 (12.3-40.0)	38.8 (30.1-45.6)

ACLD, advanced chronic liver disease; AD, acute decompensation; CI, confidence interval

Table S5. Comparison of organ dysfunctions/failures according to the CLIF-SOFA score in patients developing ACLF during follow-up between FLIS 0-3 vs. 4-6.

Patient characteristics, n (%)	FLIS 4-6, n=29	FLIS 0-3, n=28	p-value
**Liver**			
Bilirubin <6 mg/dL	16 (72.7%)	5 (18.5%)	
Bilirubin 6-12 mg/dL	1 (4.5%)	6 (22.2%)	**<0.001**
Bilirubin ≥12 mg/dL	5 (22.7%)	16 (59.3%)	
**Kidney**			
Creatinine <2 mg/dL	7 (31.8%)	7 (25.9%)	
Creatinine 2.0-3.5 mg/dL	10 (45.5%)	15 (55.6%)	0.781
Creatinine ≥3.5 mg/dL or RRT	5 (22.7%)	5 (18.5%)	
**Brain**			
West-Haven 0	11 (50.0%)	11 (40.7%)	
West-Haven I-II	5 (22.7%)	11 (40.7%)	0.397
West-Haven III-IV	6 (27.3%)	5 (18.5%)	
**Coagulation**			
INR <2.0	16 (72.7%)	16 (59.3%)	
INR 2.0-2.5	5 (17.2%)	9 (32.1%)	0.418
INR ≥2.5	3 (10.3%)	2 (7.1%)	
**Circulation**			
MAP ≥70 mmHg	14 (63.6%)	17 (63.0%)	
MAP <70 mmHg	3 (13.6%)	3 (11.1%)	0.944
Vasopressors	5 (22.7%)	7 (25.9%)	
**Respiration**			
PaO_2/FiO_2 >300	18 (81.8%)	24 (88.9%)	
PaO_2/FiO_2 200-300	1 (4.5%)	2 (7.4%)	0.428
PaO_2/FiO_2 <200	3 (13.6%)	1 (3.7%)	
**Minimal ACLF grade**			
1a	6 (27.3%)	3 (11.1%)	
1b	4 (18.2%)	5 (18.5%)	0.519
2	9 (40.9%)	15 (55.6%)	
3	3 (13.6%)	4 (14.8%)	
**Maximum ACLF grade**			
1a	5 (22.7%)	1 (3.7%)	
1b	1 (4.5%)	2 (7.4%)	0.240
2	10 (45.5%)	16 (59.3%)	
3	6 (27.3%)	8 (29.6%)	

ACLF, acute-on-chronic liver failure; FLIS, functional liver imaging score

Fig. S1

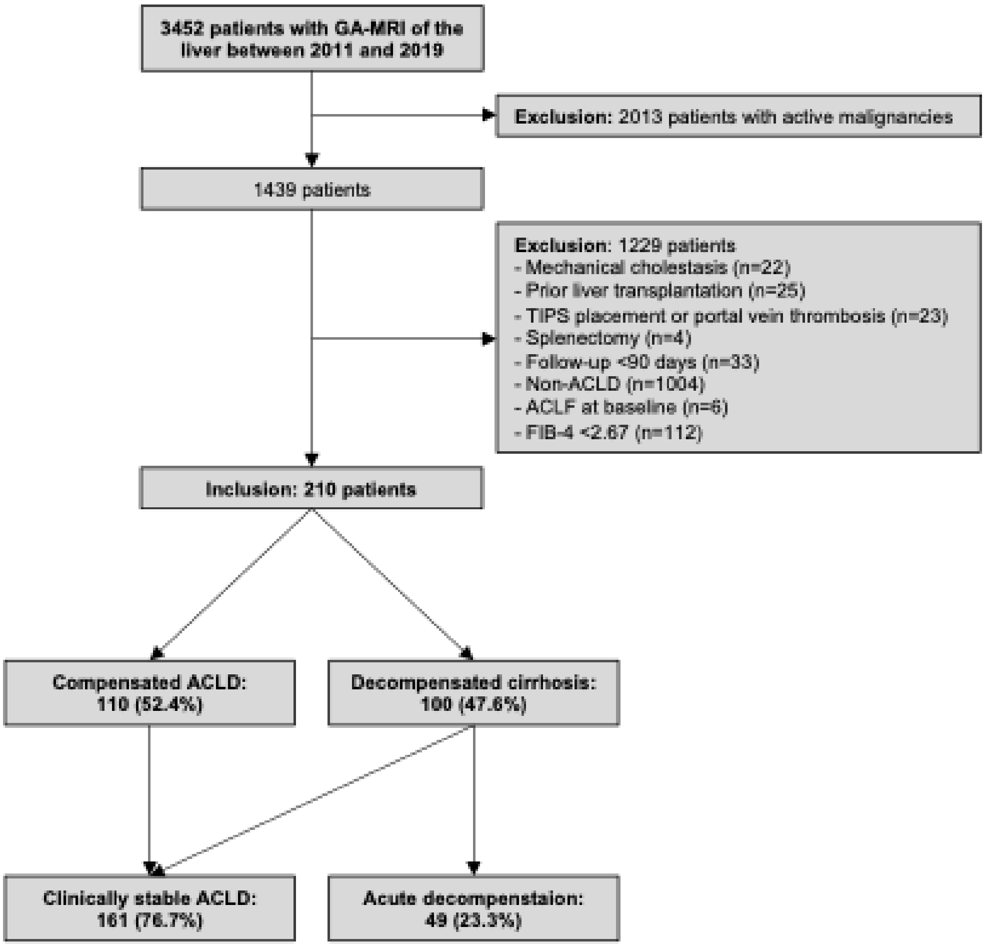

Fig. S1. Study flowchart.

Abbreviations: ACLF, acute-on-chronic liver failure; ACLD, advanced chronic liver disease; AD, acute decompensation; cACLD, compensated advanced chronic liver disease; GA, gadoxetic acid; MRI, magnetic resonance imaging; TIPS, transjugular intrahepatic portosystemic shunt
